# Venom Composition of Neglected Bothropoid Snakes from the Amazon Rainforest: Ecological and Toxinological Implications

**DOI:** 10.3390/toxins16020083

**Published:** 2024-02-04

**Authors:** Luciana A. Freitas-de-Sousa, Mônica Colombini, Vinicius C. Souza, Joanderson P. C. Silva, Ageane Mota-da-Silva, Marllus R. N. Almeida, Reginaldo A. Machado, Wirven L. Fonseca, Marco A. Sartim, Jacqueline Sachett, Solange M. T. Serrano, Inácio L. M. Junqueira-de-Azevedo, Felipe G. Grazziotin, Wuelton M. Monteiro, Paulo S. Bernarde, Ana M. Moura-da-Silva

**Affiliations:** 1 Laboratório de Imunopatologia, Instituto Butantan, São Paulo 05503-900, SP, Brazil; luciana.sousa@butantan.gov.br (L.A.F.-d.-S.); monica.colombini@butantan.gov.br (M.C.); 2 Laboratório de Toxinologia Aplicada, Instituto Butantan, São Paulo 05503-900, SP, Brazil; vinicius.souza@esib.butantan.gov.br (V.C.S.); joanderson.psilva@butantan.gov.br (J.P.C.S.); solange.serrano@butantan.gov.br (S.M.T.S.); inacio.azevedo@butantan.gov.br (I.L.M.J.-d.-A.); 3 Instituto Federal do Acre, Campus de Cruzeiro do Sul, Cruzeiro do Sul 69980-000, AC, Brazil; ageane.silva@ifac.edu.br; 4 Laboratório de Herpetologia, Universidade Federal do Acre, Campus Floresta, Cruzeiro do Sul 69895-000, AC, Brazil; marllus.almeida@sou.ufac.br (M.R.N.A.); reginaldo.machado@ufac.br (R.A.M.); wirven.fonseca@ufac.br (W.L.F.); paulo.bernarde@ufac.br (P.S.B.); 5 Instituto de Pesquisa Clínica Carlos Borborema, Fundação de Medicina Tropical Dr. Heitor Vieira Dourado, Manaus 69040-000, AM, Brazil; marcosartim@hotmail.com (M.A.S.); jac.sachett@gmail.com (J.S.); wueltonmm@gmail.com (W.M.M.); 6 Laboratório de Coleções Zoológicas, Instituto Butantan, São Paulo 05503-900, SP, Brazil; felipe.grazziotin@butantan.gov.br

**Keywords:** snake venoms, transcriptomics, proteomics, antivenom, Amazon, *Bothrops*, *Bothrocophias*

## Abstract

Snake venoms have evolved in several families of Caenophidae, and their toxins have been assumed to be biochemical weapons with a role as a trophic adaptation. However, it remains unclear how venom contributes to the success of venomous species for adaptation to different environments. Here we compared the venoms from *Bothrocophias hyoprora*, *Bothrops taeniatus*, *Bothrops bilineatus smaragdinus*, *Bothrops brazili*, and *Bothrops atrox* collected in the Amazon Rainforest, aiming to understand the ecological and toxinological consequences of venom composition. Transcriptomic and proteomic analyses indicated that the venoms presented the same toxin groups characteristic from bothropoids, but with distinct isoforms with variable qualitative and quantitative abundances, contributing to distinct enzymatic and toxic effects. Despite the particularities of each venom, commercial *Bothrops* antivenom recognized the venom components and neutralized the lethality of all species. No clear features could be observed between venoms from arboreal and terrestrial habitats, nor in the dispersion of the species throughout the Amazon habitats, supporting the notion that venom composition may not shape the ecological or toxinological characteristics of these snake species and that other factors influence their foraging or dispersal in different ecological niches.

## 1. Introduction

Almost 435 species of snakes are known to inhabit the Brazilian territory, of which 238 occur in the Amazon Forest [1] and 80 have already been recorded in the Alto Juruá region at Acre, the westernmost state of Brazil [2]. The Alto Juruá region still maintains more than 80% of its original vegetation with high complexity and environmental heterogeneity [3]. It is characterized by a dendritic system of water courses [4] shared between Peru and the Brazilian states of Acre and Amazonas. Recently, Bernarde and collaborators recorded venomous species in the region that are included in the Elapidae and Viperidae families. In Viperidae, the presence of *Crotalus* spp. was not detected, but *Lachesis muta* (bushmaster) and bothropoids (lanceheads) were frequent. Among the bothropoids, five species were recorded: *Bothrocophias hyoprora*, *Bothrops atrox*, *Bothrops bilineatus smaragdinus*, *Bothrops brazili*, and *Bothrops taeniatus* [2]. They are all endemic species of the Amazon, extending to eastern Ecuador, southern Colombia, Venezuela, northwestern Peru and Bolivia, Guyana, Suriname, and French Guiana [2]. Although sympatric and with a mainly generalist feeding habit, these species differ in terms of their activity substrates and the population sizes in the territories they occupy, with the consequent implications for snakebites [2]. *B. b. smaragdinus* and *B. taeniatus* are arboreal species in contrast to *B. hyoprora*, *B. brazili*, and *B. atrox*, which are predominantly terrestrial [2]. However, the most notable point is the abundance in which *B. atrox* occupies the entire territory of the Amazon, being the main cause of snakebites in the region [5]. The composition of the venom of these snakes could be one of the factors favoring the differences in activity substrates and population dispersal in different habitats. Venom represents an adaptive trophic trait, and venom variation is shaped by a variety of evolutionary processes and ecological adaptations such as diet [6,7,8], environmental conditions [9], snake ontogeny [10,11,12,13,14,15], and geographical distribution [13,16,17,18]. Therefore, snake venoms provide an ideal system for investigating the mechanisms shaping the snake ecological adaptations with the great advantage of displaying clear genotype–phenotype relationships and measurable bioactive properties.

There are many studies describing the composition and variability of *B. atrox* venom from specimens collected in different geographical areas and habitats of the Amazon [12,19,20,21,22]. In opposition, only a few papers report the composition of venoms from *B. brazili* [23], *B. b. smaragdinus* [24], and *Bothrocophias* spp. [25,26,27], and little is known about the venom composition of *B. taeniatus* [27]. Interestingly, these reports indicate similar characteristics among the venoms of these species comprising toxins belonging to the most characteristic groups from *Bothrops* snake venoms including snake venom metalloproteinases (SVMPs), snake venom serine proteinases (SVSPs), phospholipases A_2_ (PLA_2_), and C-type lectin-like proteins (CTL), majorly. However, the available data were acquired from specimens collected from different locations, mostly from captivity, and different ontogenetic stages. Such conditions are known to interfere with the abundance of each toxin group in the venoms making it difficult to correlate venom composition with the different ecological characteristics of the snake species. Thus, here we sought to describe and compare the venom gland transcriptomes and venom proteomes from specimens of *B. hyoprora*, *B. taeniatus*, *B. b. smaragdinus*, *B. brazili*, and *B. atrox* collected in the Alto Juruá region, aiming to address the possible role of venom composition and toxinological properties in shaping the ecological characteristics of each species.

## 2. Results and Discussion

**Figure 1:**
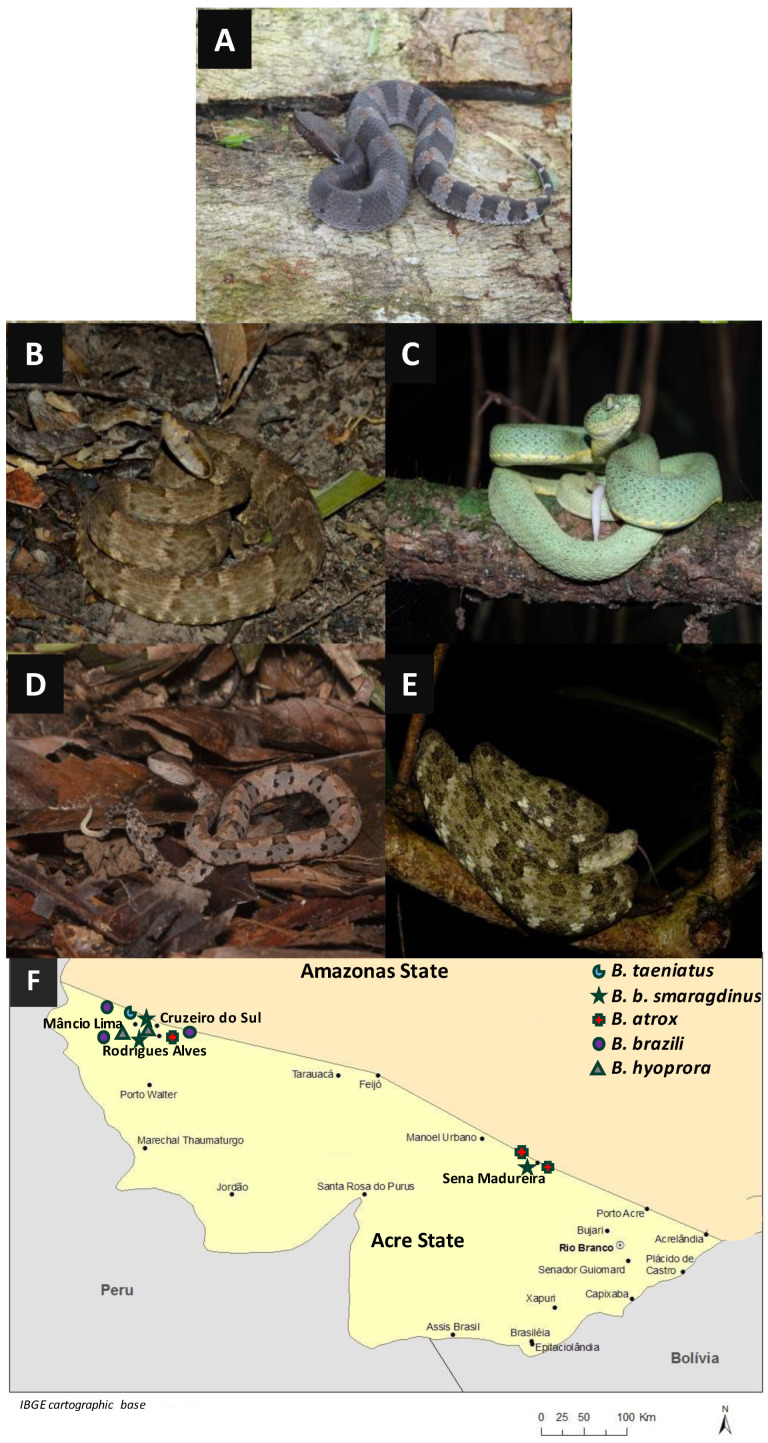
Representative specimens of the species used in this study: *Bothrocophyas hyoprora* (**A**); *B. atrox* (**B**); *B. bilineatus smaragdinus* (**C**); *B. brazili* (**D**); *B. taeniatus* (**E**); and geographical distribution of the collection areas (**F**). Pictures, (**A**,**D**): PS Bernarde; (**C**,**F**): WL Fonseca; and (**E**): MRN Almeida.

To examine the venom composition of snakes, we selected two specimens of *Bothrocophias hyoprora*, three specimens of *B. b. smaragdinus*, three specimens of *B. brazili*, three specimens of *B. atrox*, and one specimen of *B. taeniatus*, illustrated in Figure 1A–E, collected in the Acre State of Brazil, mostly in the Alto Juruá region (Figure 1F).

**Figure 2:**
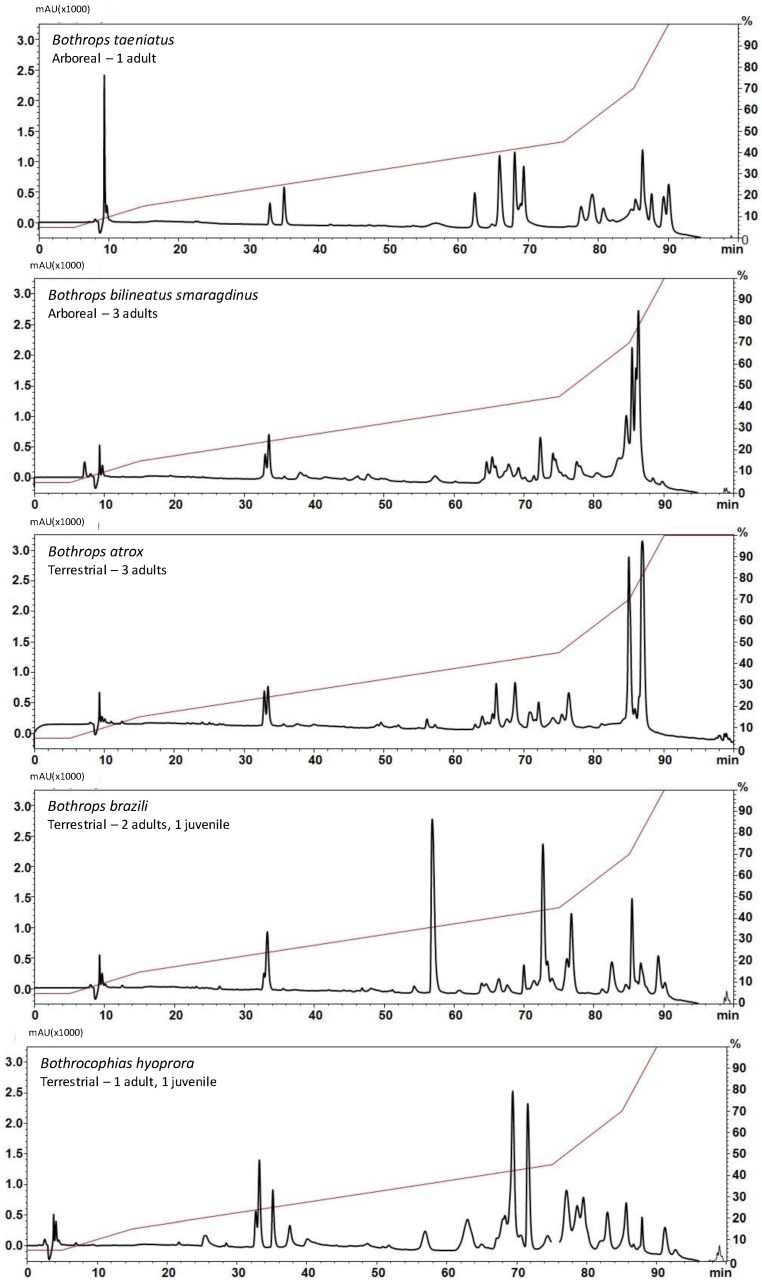
Comparison of the chromatographic profiles of venom samples from *B. taeniatus*; *B. bilineatus smaragdinus*; *B. atrox*; *B. brazili*; and *Bothrocophyas hyoprora*: Individual venom samples (2 mg) were applied to a C-18 column. Mobile phases used were 0.1% TFA in water (solution A) or 0.1% TFA in acetonitrile (solution B). Proteins were gradient eluted at 2 mL/min as depicted by the red line. Separation was monitored at 214 nm.

As a first step of the comparison, venoms were pooled according to the snake species and subjected to reversed-phase high-performance liquid chromatography (RP-HPLC) chromatography under standard conditions [9]. As shown in Figure 2, the elution profiles were overall consistent with the fractionation of bothropoid venoms under similar conditions [27,28,29,30]; however, the size of the peaks eluted in similar retention times varied considerably suggesting the different quantitative distribution of toxin isoforms amongst the species. Peaks eluted after 80 min, which are correlated to SVMPs and frequent in venoms of *Bothrops* snakes [9], were characteristic in *B. atrox*, *B. taeniatus*, and *B. b. smaragdinus* snake venoms. Another marked position in the chromatograms is the peak corresponding to noncatalytic K49-PLA_2_ homologs eluted between 55 and 60 min, detected in higher abundance in the *B. brazili* venom chromatogram while much lower absorbances were detected in the other venom chromatograms at the same elution time. *B. hyoprora*, *B. taeniatus*, and *B. brazili* venoms showed a very complex profile between 60 and 80 min, which usually elutes different toxins as SVSPs, CTLs, acidic PLA_2_s, and other minor components [9].

To understand the venom complexity, we decided to compare the amino-acid sequences of toxin isoforms and their expression levels in the biochemical arsenal produced by each species. Thus, we first sequenced, assembled, and characterized the venom gland transcriptome of selected snakes, followed by a shot-gun proteomic analysis and functional assays of the venom pools.

Concerning the transcriptomes, the number of merged reads ranged from approximately 4,000,000 in *B. b. smaragdinus* to 30,000,000 in *B. hyoprora*. Reads were assembled into contigs yielding an average of 173,981 contigs per species, from which 540, on average, were predicted as putative toxins before curation (Supplementary Table S1).

After annotation, the number of recovered toxins and toxin families (Table 1) was generally consistent with those of other viperid venom gland transcriptomes [31,32,33,34,35,36]. We annotated 264 transcripts of predicted toxins for the five species, which corresponded to fully coding sequences of 19 toxin families. SVMPs (Snake Venom Metalloproteinases), CTL (C-Type Lectin-Like Toxins), SVSP (Snake Venom Serine Proteinases), PLA_2_ (Phospholipases A_2_), and VEGF (Vascular Endothelial Growth Factors) were represented by multiple independent transcripts in venoms from the five species. CRISPs (Cysteine Rich Secretory Proteins), PDEs (Phosphodiesterases), and PLBs (Phospholipases B) were detected in all venom glands, but mostly as a single copy. LAAOs (L-Amino Acid Oxidases), HYAL (Hyaluronidases), NGF (Nerve Growth Factors), NUCs (5′Nucleotidases), BPPs (Bradykinin Potentiating Factors), DIPEP (Dipeptidyl Peptidases), CYS (Cystatins), iPLAs (Phospholipase inhibitors), LIPAs (Lipases), WAP (Warprins), and KUN (Kunitz Trypsin Inhibitors) were not detected in all venom samples, or if detected, it was as a single copy (Table 1).

**Figure 3:**
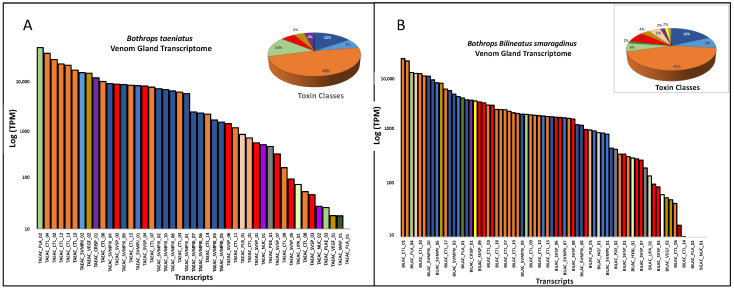

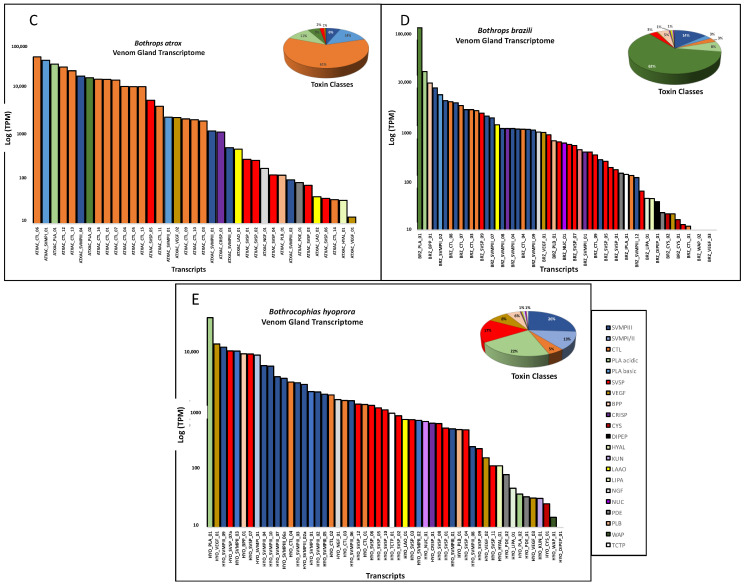
Venom gland transcriptomes: *B. taeniatus* (**A**); *B. bilineatus smaragdinus* (**B**); *B. atrox* (**C**); *B. brazili* (**D**); and *Bothrocophyas hyoprora* (**E**). Bar plots and pie charts represent the average venom composition of each species classified by toxin class.

In Figure 3 we show the summary of venom gland transcriptomes from the five species. The venom transcriptomes of *B. taeniatus*, *B. b. smaragdinus*, and *B. atrox* are characterized by the high expression of CTLs, accounting for 48.6%, 44.0%, and 60.4%, respectively, of total toxin transcripts (Figure 3A–C). The transcriptome of the *B. brazili* venom gland was largely dominated by PLA_2_s (67.13%), mostly represented by a single transcript encoding a K49 noncatalytic homologue of snake venom PLA_2_s (59.46%) (Figure 3D). In the *B. hyoprora* venom gland transcriptome, the most abundant toxin groups were the SVMPs (39.2%) and PLA_2_s (22.2%), the latter represented by a single transcript of an acidic PLA_2_ isoform (Figure 3E). PLA_2_s presented a low transcript number, but they are among the highest expressed in all species. Some of the SVMP transcripts also figure as the most expressed, even in CTL-rich transcriptomes (Figure 3).

It is important to point out that transcriptomes were carried out individually and the data presented here are the mean of TPMs obtained in each species. Some cases of quantitative intraspecific variation were observed comparing the transcript abundances: two isoforms of SVMP and two of SVSP were more abundant in the *B. hyoprora* adult snake, but these transcripts were not represented in the proteomes; quantitative and qualitative higher abundance in CTL isoforms were observed in the *B. b. smaragdinus* collected in Resex Cazumbá, Sena Madureira, which could be attributed to geographical or habitat variation; adult specimens of *B. brazili* presented proportionally higher amounts of PLA_2_, indicating ontogenetic variation. However, the interpretation of these data is beyond the scope of this manuscript.

To our knowledge, transcriptomes from *B. hyoprora*, *B. taeniatus*, *B. b. smaragdinus*, and *B. brazili* venom glands have so far not been described. In contrast, *B. atrox* venom gland transcriptomes have been described by Neiva and collaborators [37], using expressed sequence tags (ESTs), and by our group [31], via Next Generation Sequencing using Illumina technology. Interestingly, the transcriptomic analysis via these methods resulted in a different pattern compared to the one described here. In both studies, SVMPs dominated the transcriptomes with expression levels higher than 60% while the expression of CTL transcripts was lower, comprising 5 to 20% of total toxin expression. The divergences between these data and the data from Neiva and collaborators [37] could be attributed to the different methodologies used in both studies. However, in our previous study [31] we used similar methods for sequencing and assembling the contigs, suggesting that the differences observed could be due to intraspecific variation as the geographical distance between the places in which snakes were collected could be a likely variable for explaining the higher proportion of CTLs (60.4%) and lower expression of SVMPs (20.2%) described in this study.

**Figure 4:**
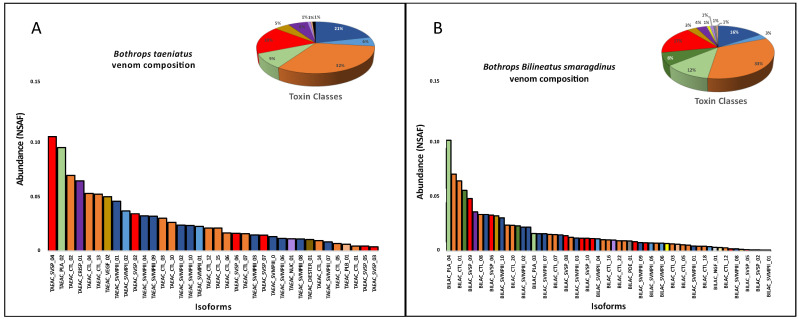

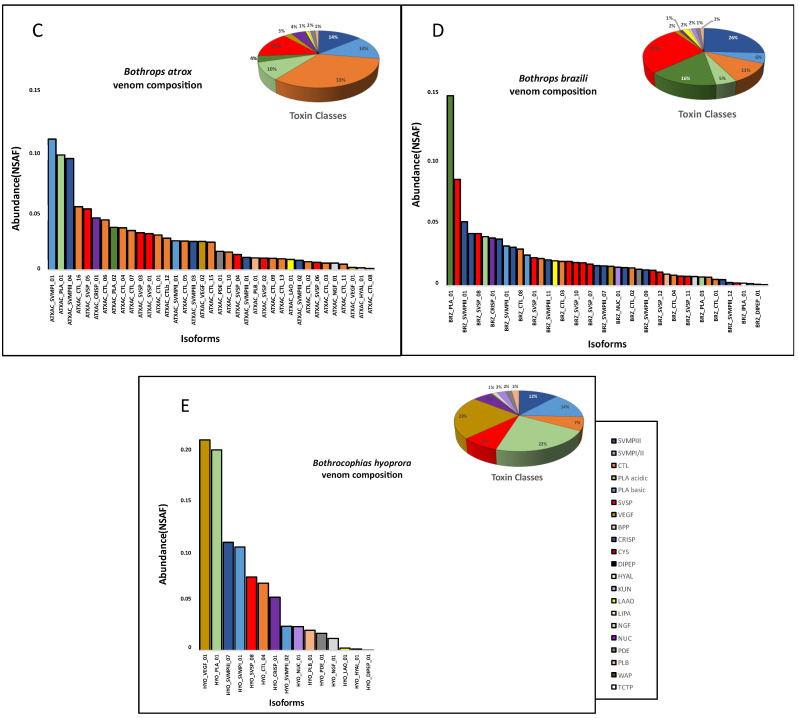
Distribution of protein groups and isoforms in venom proteomes: *B. taeniatus* (**A**); *B. bilineatus smaragdinus* (**B**); *B. atrox* (**C**); *B. brazili* (**D**); and *Bothrocophyas hyoprora* (**E**). Bar plots and pie charts represent the average venom composition of each species run in triplicates, classified by toxin class. Relative expression is indicated as Normalized Spectral Abundance Factor (NSAF) quantitative method of Scaffold 5.0.

The venom proteomic analysis (Figure 4) was carried out using pooled venoms from each species. Even considering the individual differences observed in the transcriptomes, the proteomic and functional characterization of the venoms could not be carried out individually due to the minor quantities of venom collected from a single snake. Spectra identification and quantification were based on the protein sequences predicted from a non-redundant transcriptome master set of the same specimens used for venom extraction. As observed in the venom gland transcriptomes, in venom proteomes CTLs were the most abundant toxin group in the venoms of *B. taeniatus* (31.6%), *B. b. smaragdinus* (33.0%), and *B. atrox* (32.0%) while SVMPs and PLA_2_s dominated the venoms of *B hyoprora* (23.7% and 20.2%) and *B. brazili* (20.2% and 20.4%). Interestingly, only PLA_2_ acidic isoforms were present in *B. hyoprora* venom while non-catalytic PLA_2_ basic homologues are the major isoform in *B. brazili* venom. Comparing the abundance of toxin groups detected in the proteomes and transcriptomes, in *B. taeniatus*, *B. b. smaragdinus*, and *B. atrox* venoms we observed a marked decrease in the relative amounts of CTLs (average of 51.3% in transcriptomes to 32.2% in proteomes) and a decrease in the relative expression of PLA_2_s in *B. brazili* venom (62% in the transcriptome to 35% in the proteome). In opposition, a relevant increase in the relative abundance of SVSPs was observed in the proteomes of all venoms with an average of 7.3% in transcriptomes, rising to 16.4% in proteomes (Figure 3 and Figure 4). This finding may be explained by differences in the ionization of trypsin fragments from each toxin group, but differences in translation rates of transcripts from each toxin type may not be disregarded. In our previous study [31], we observed that many transcripts, mostly of CTLs, were not translated to the corresponding venom isoform, even those with high transcriptional rates. Moreover, a similar observation was reported by Mason and collaborators [38] showing intraspecific variation in transcript expression levels between two specimens of *B. nigroviridis*.

There are several studies concerning the venom composition, variability, and functional properties of *B. atrox* venom [12,39,40,41,42]. However, only a few reports are available concerning the other venoms. Proteomes of *B. brazili* [23] and *B. b. smaragdinus* [24] have already been reported. The venom composition previously reported for *B. brazili* was very similar to the results reported in this study, characterized by the marked presence of basic PLA_2_ K49 homologs [23]. Interestingly, the previous study was performed with pooled venoms from captivity specimens originally from the Brazilian Pará State (eastern Amazon) while our study used venoms from wild specimens collected in Acre (western Amazon). The similarity between the venom composition observed in both studies suggests that the geographical distance between the two localities or the conditions in which snakes were maintained do not appear to modulate the composition of *B. brazili* venom. However, the opposite was observed comparing the previous venom proteomes of *B. b. smaragdinus*. In the previous study [24], SVMPs were the most abundant toxin group followed by lower proportions of CTLs, distinct from our data that showed the CTLs as the dominant toxin group. Here, all snakes analyzed were collected in the western Amazon, while in the previous study, proteomes were generated based on two sets of pooled venoms, as follows: (1) from two snakes born in captivity from a pregnant female collected in the western Amazon; and (2) from venoms extracted from 10 specimens collected in Peru. Both sets of pooled venoms show SVMP dominance and low levels of CTL, particularly in the Peruvian specimens [24]. At first glance, these data suggests that venom variability is higher in *B. b. smaragdinus* than in *B. brazili* snakes. However, the methods of isoform quantification used in these studies are different; therefore, this observation should be taken with caution. On the other hand, compared to previous studies, we observed in this study a reduction in SVMP abundance and an increase in CTL in the venoms of *B. atrox*. In previous studies carried out by our group, we observed a higher abundance of SVMPs in venoms of *B. atrox* snakes collected in the Pará State (eastern Amazon) [31], Amazonas State (central Amazon), and Maranhão State (western Amazon) [43]. In these previous studies, proteomics quantification has been carried out with the same methods used here (shot-gun proteomic analysis and protein quantification based on the species transcriptome); therefore, it is reasonable to assume that the composition of *B. atrox* venoms from specimens collected in Acre is indeed richer in CTLs rather than in SVMPs, and the same may be acceptable for *B. b. smaragdinus*. Proteomes of *B. hyoprora* and *B. taeniatus* venoms have not been described so far, and venom composition has been inferred via the C18 RP-HPLC chromatographic profiles of venoms from these species collected in Peru [27]. The RP-HPLC profile of *B. taeniatus* venom was similar to the one reported in this study, suggesting a small variation in venom composition between the specimens involved in both studies.

Important differences were noted in *B. hyoprora* chromatograms, particularly by the presence of the K49-PLA_2_ homologs described previously that were not detected here with the samples collected in Acre. The presence of K49-PLA_2_ homologs has also been reported in proteomes of *Bothrocophias campbelli* [26] and *Bothrocophias myersi* [25] venoms. Thus, we attempted to search the spectra generated in the LC-MS/MS using the *B. hyoprora* venom pool against a comprehensive databank comprising 111,653 sequences from snake venom-related proteins [44]; and even in this search, K49-PLA_2_ isoforms were not detected in the *B. hyoprora* venom proteome, evidencing their absence in the venom of *B. hyoprora* from Acre.

**Figure 5:**
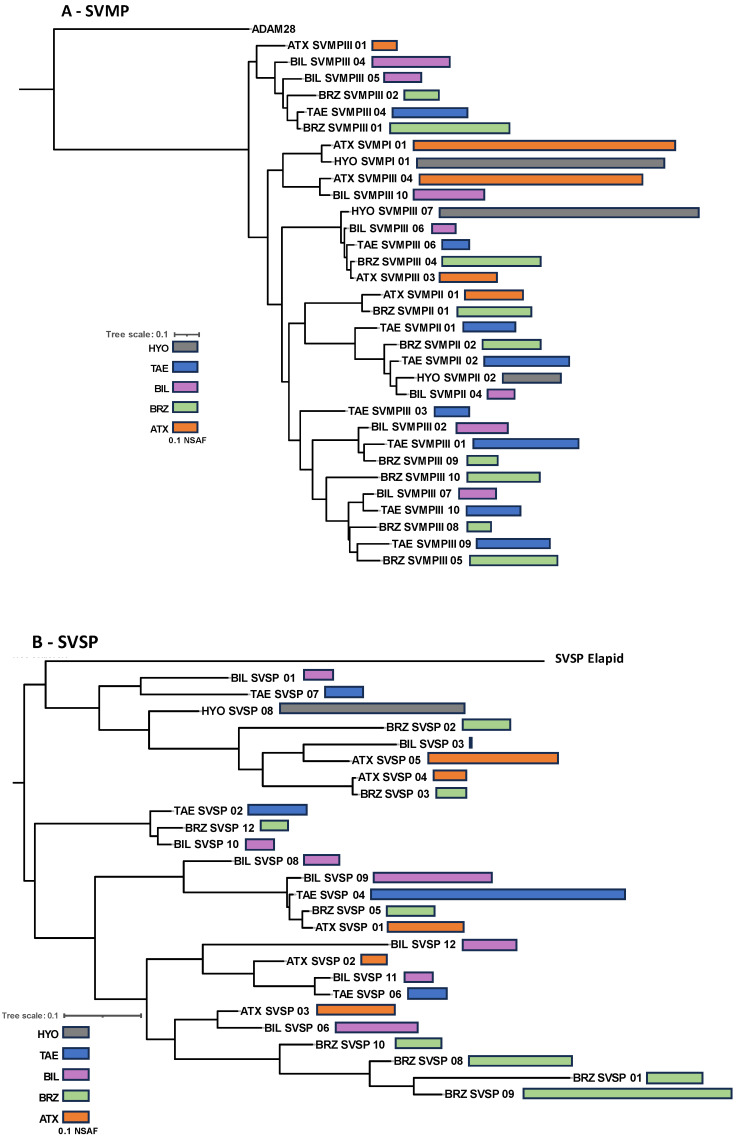

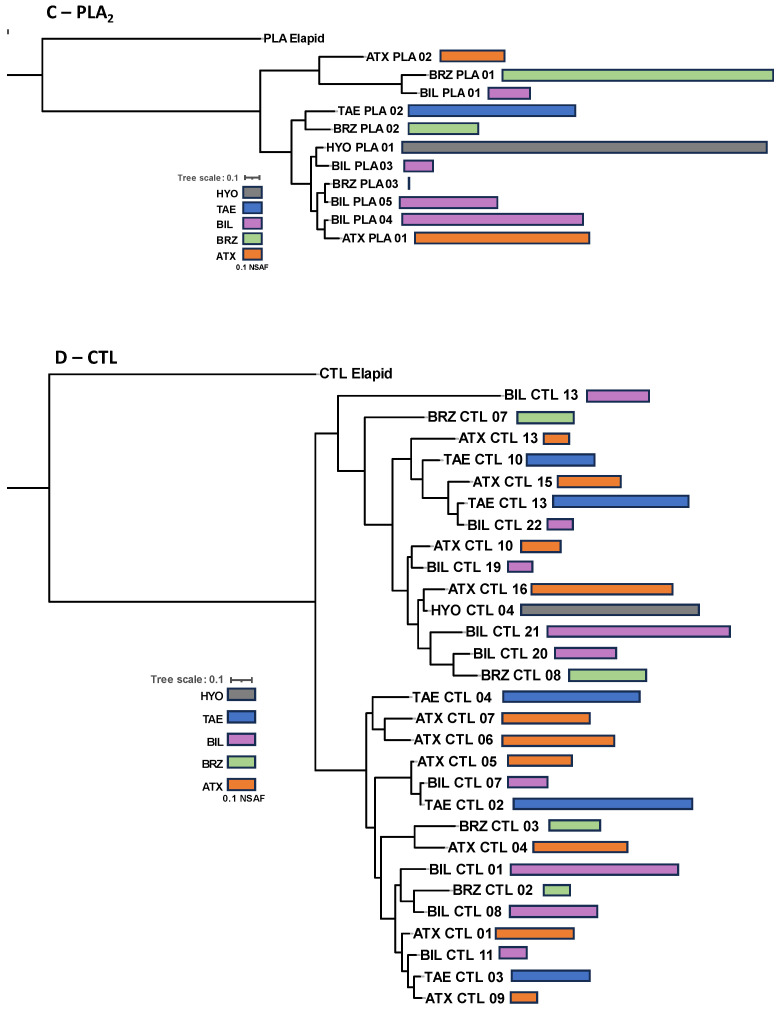
Phylogenetic inferences of isoforms from the most abundant toxin groups: Complete deduced protein sequences of SVMPs (**A**), SVSPs (**B**), PLA_2_s (**C**), and CTL (**D**) were aligned using MAFF-T. The maximum likelihood phylogenetic tree was generated using PhyML. Bars represent the quantitative value (NSAF) of the isoforms detected via LC-MS/MS in each venom sample, which are represented by the different colors of the bars, as indicated in the figure.

In summary, the venom composition of the five species studied conserved the same protein families present in venoms of bothropoid snakes, but with important quantitative differences in the proteomes and the chromatographic profiles. This suggests that the presence of different isoforms representing the main protein families in each venom may account for the qualitative differences observed in the chromatograms. Therefore, in the next step, we aligned the primary sequences and compared the relative expression (NSFA) of the most abundant isoforms of SVMPs, PLA_2_s, CTLs, and SVSPs present in the venoms. In the phylogenetic analysis of each toxin group (Figure 5), *B. hyoprora* venom appeared to be the least complex, presenting only one sequence of CTL, one of SVSP, and one of PLA_2_ that clustered with similar isoforms from venoms of the other species. Two SVMP isoforms were present in the venom, one of a PI-class that showed 94.4% identity with Atroxlysin-Ia, a hemorrhagic PI-class SVMP widely represented in different samples of *B. atrox* venom [45,46], and one PIII-class SVMP that clustered with similar isoforms from venoms of the other species. This suggests that the phylogenetic distances among the species, as described by Alencar and collaborators [47], are not represented in the diversification of the venom toxins. It is important to note that the *B. hyoprora* transcriptome revealed a more complex pattern on transcripts with 14 different SVMPs, 12 SVSPs, 4 CTLs, and 2 PLA_2_s. The reason why only a few transcripts were detected or translated to the venom is still unclear. The venoms of the other species shared a higher complexity, with several different isoforms that clustered with their homologs present in the other species, suggesting that ancestor ortholog genes are similarly active in all species. However, some peculiarities were also observed. The highest expression of two SVMP isoforms, from PI- and PIII-classes, was in *B. atrox* venom (Figure 5A); the presence of one cluster of SVSPs appeared to be exclusive to *B. brazili* venom (Figure 5B); and the presence of the K49-PLA_2_ homologs were only found in *B. b. smaragdinus* and *B. brazili* venoms (Figure 5C).

**Figure 6:**
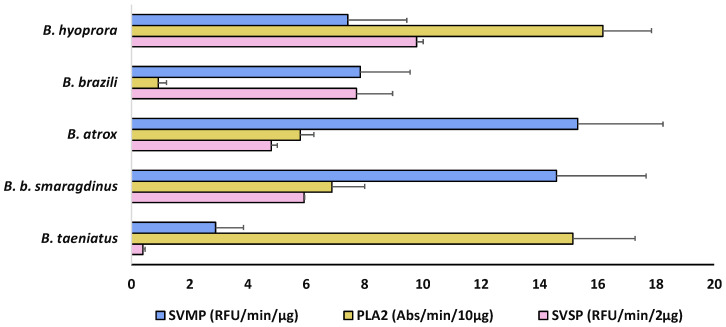
Enzymatic activities of *B. taeniatus*, *B. bilineatus smaragdinus*, *B. atrox*, *B. brazili*, and *Bothrocophyas hyoprora* venoms: SVMP catalytic activity was evaluated via hydrolysis of a FRET substrate (Abz-AGLA-EDDnp), and results are expressed as RFU/min/µg of venom. SVSP catalytic activity was evaluated via hydrolysis of the chromogenic synthetic substrate (L-BAPNA) and expressed in Abs/min/2 µg of venom. PLA_2_ activity was evaluated via hydrolysis of the chromogenic substrate (NOBA) and the results are expressed in Abs/min/10 µg of venom. The data shown represent the mean + SD of three independent experiments.

**Figure 7:**
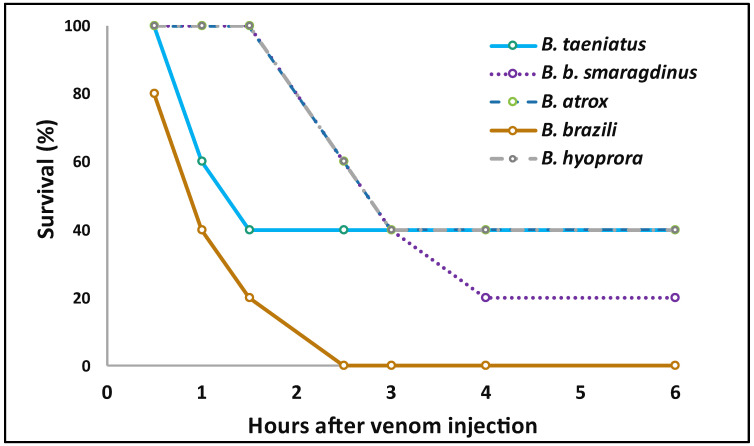
Lethality of *B. taeniatus*, *B. bilineatus smaragdinus*, *B. atrox*, *B. brazili*, and *Bothrocophyas hyoprora* venoms in murine models: Single doses of venom (300 μg) were injected i.p. into groups of five mice. Next, the deaths were followed up every hour until 6 h after, then at 12, 24, and 48 h after venom injection. The graphic shows the *X* axis only up to 6 h, as no death was recorded after this time.

The presence and abundance of different isoforms result in the overall biological effects of the venoms. Indeed, in Figure 6, we show a comparison of the enzymatic activities of each pool of venom. SVMP activity varied among the venoms and was highest in the venoms of *B. atrox* and *B. b. smaragdinus* (Figure 6, blue bars), which could be explained by the presence of ATXSVMPIII-04 and BILSVMPIII-10 isoforms amongst the most expressed SVMPs of *B. atrox* and *B. b. smaragdinus*, respectively. These two isoforms present similar primary structures sharing more than 90% identity with Jararhagin and Batroxrhagin [48], known to be multifunctional hemorrhagic metalloproteinases, conserved in venoms of several species of vipers [31]. *B. hyoprora* and *B. taeniatus* presented the highest PLA_2_ activity (Figure 7, yellow bars), probably due to the high catalytic activity of acidic PLA_2_, dominant in both venoms. In opposition, *B. brazili* venom presented the highest content of PLA_2_ isoforms but a very low catalytic activity, which could be attributed to the lack of catalytic activity in the K49-PLA_2_ homologs abundant in this venom [49]. Considering SVSPs, *B. brazili* venom contained a high catalytic activity, which agrees with the highest abundance of SVSPs in the venom proteome. However, *B. hyoprora* had the lowest proportion of SVSPs in the venom proteome but presented the highest activity indicating a high specific activity of the single isoform expressed in the venom. Interestingly, *B. taeniatus* venom contained a very low catalytic SVSP activity, which agrees with the lack of procoagulant [50] or defibrinating [51] activities previously reported for this venom.

All species studied here present a generalist diet in their first ontogenetic stages, feeding on small ectothermic prey, changing to a more specialist diet when they became larger and mainly prey on small rodents [52]. To estimate the lethality of these venoms we decided to use the percent survival time of experimental mice injected with lethal amounts of venom. Usually, venoms of *Bothrops* species present LD_50_ values below 100 µg venom/mice [53]. Thus, we initially injected groups of five mice with 100 µg venom/mice. Interestingly, the lethality of the tested venoms in the experimental mice was not high and no animal died with such dose. Injection of 300 μg of venoms of *B. hyoprora*, *B. taeniatus*, *B. atrox*, and *B. b. smaragdinus* was partially sufficient to promote the death of the experimental mice over 48 h. In such conditions, only *B. brazili* venom promoted the death of all animals in less than 3 h (Figure 7). Unfortunately, due to the limited amount of venom available and the high quantity used in the test, the experiment was not repeated, giving only an indication that *B. brazili* venom could be more lethal to mice. The differences in *B. brazili* venom that could correspond to its higher lethality are not evident. However, it is reasonable to assume that the higher amounts of K49-PLA_2_s and SVSPs in *B. brazili* venom could favor its higher lethal activity. High concentrations of myotoxic PLA_2_s have been related to the rapid immobilization of mammals, preventing them from moving away [54], while procoagulant SVSPs participate in the impairment of hemostasis in mammalian and avian prey [55]. Moreover, the neutralization of *B. asper* venom lethality in mice correlated with the neutralization of in vitro coagulant activity, not necessarily involving the SVMPs [56]. Thus, SVSPs and myotoxic PLA_2_s venom could correspond to the higher lethality in the experimental mice in *B. brazili*. Interestingly, *B. atrox* snakes that show the widest distribution in all the Amazon Forest have venoms with the least capacity to kill small rodents, and this is not a feature of *B. atrox* from Alto Juruá since the venoms of snakes collected in different habitats of the Pará state also presented high LD_50_ doses [9].

Among the species studied here, *B. atrox* is responsible for most human snakebite envenoming in the Amazon region [57,58] followed by *B. b. smaragdinus*, which is involved in human accidents caused mainly during activities within forests, such as the opening up of new trails or collecting fruits of the acai palm. Snakebites by *B. brazili*, *B. taeniatus*, and *B. hyoprora* are rarely reported but their potential harm to humans should not be ignored. Although accidents with these species are not frequent, some clinical signs could be foreseen based on the toxins detected by proteomics. The toxin groups detected here match the composition of venoms from different species of *Bothrops*, suggesting that in human accidents with the species studied here, there will be potential local and systemic signs and symptoms related to coagulant activities and hemorrhagic and inflammatory effects [50,59]. Indeed, a single report of a human accident with *B. taeniatus* described that the patient developed a clinical picture compatible with mild envenomation by *B. atrox* with small bleeding at the bite site and edema just below the knee without development of coagulopathy [60]. Also, even when collected outside the Brazilian territory, the activities of the *B. bilineatus* and *B. taeniatus* venoms are dominated by high proteolytic action on thrombin, plasmin, kallikrein, and cathepsin C [61]. Predicting signs of envenoming according to the abundance of toxin groups, the presence of basic PLA_2_s indicates the possibility of severe muscle damage in patients bitten by *B. brazili* and very low myotoxicity in patients bitten by *B. hyoprora* or *B. taeniatus*. On the other hand, the acidic PLA_2_ isoforms dominant in *B. hyoprora* or *B. taeniatus* venom predict anticoagulant activity in these venoms. In addition, the high proportions of CTL in the venoms of *B. b. smaragdinus*, *B. atrox*, and *B. taeniatus* collected in Acre indicates hemostatic disturbances involving platelet activation [62] that may result in their depletion aggravated by anticoagulation as CTLs may be potent inhibitors of thrombin [63,64,65].

**Figure 8:**
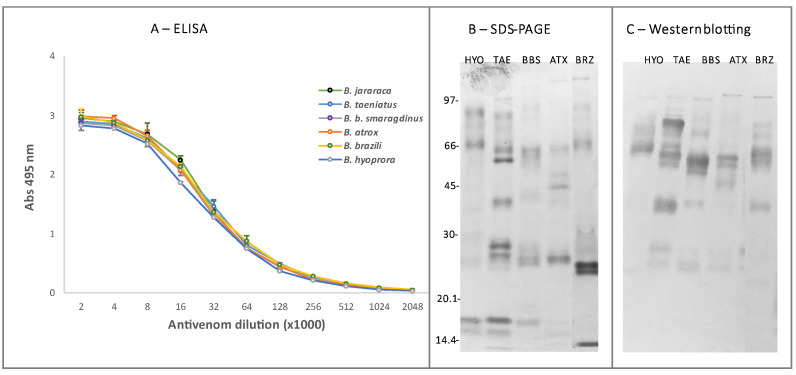
Reactivity of *B. taeniatus* (TAE), *B. bilineatus smaragdinus* (BBS), *B. atrox* (ATX), *B. brazili* (BRZ), *and Bothrocophyas hyoprora* (HYO) venoms with *Bothrops* commercial antivenom: For ELISA (**A**), venom samples (5 μg/mL) were coated to microplates (Nunc) and incubated with crescent dilutions of Bothrops commercial antivenom (starting from 1:2000), followed by incubation with anti-horse IgG labeled with peroxidase (1:2000). The reactions were developed with ortho-phenylenediamine/H_2_O_2_ as the enzyme substrate, and the products were detected at 490 nm. The experiment was repeated twice, and the Abs represents the mean ± SD of the two experiments. Next, venoms were fractionated via SDS-PAGE in 12.5% acrylamide gel under nonreducing conditions and were either stained with Coomassie blue (**B**) or transferred to nitrocellulose membranes, which were then incubated with the antivenom (1:2000) as the primary antibody and peroxidase-labeled goat anti-horse IgG (1:2000). The reactive bands were detected via incubation with 4-chloro-a-naphthol and H_2_O_2_ (**C**). The numbers at the left indicate the mobility of the molecular mass markers in kDa.

The recommended treatment for snakebites is the use of heterologous antivenoms. Based on the similarity among venoms of *Bothrops* snakes, commercial antivenom in Brazil is produced via horse immunization with a pool of *Bothrops* venoms (*Bothrops alternatus*, *Bothrops jararaca*, *Bothrops jararacussu*, *Bothrops moojeni*, and *Bothrops neuwiedi*) [66] that does not include venoms of any species from the Amazon. Therefore, we tested the reactivity and neutralization of the commercial *Bothrops* antivenom produced in Brazil against the venoms of *B. b. smaragdinus*, *B. atrox*, *B. brazili*, *B. taeniatus*, and *B. hyoprora* snakes. As shown in Figure 8A, similar ELISA titration curves of the antivenom were observed in plates coated with the different venoms from Acre and against *B. jararaca* venom, which is the major immunogen included in the production of *Bothrops* antivenom in Brazil. Accordingly, via Western blotting we observed that the commercial antivenom recognized bands of higher molecular masses in all venoms; however, the recognition of bands below 20 kDa was feeble (Figure 8C). Despite that, SAB completely neutralized the lethality of all tested venoms. Similar data were reported by Muniz and coworkers [67], showing that the commercial antivenom preferentially recognized components with a relative mass above 66 kDa and that it neutralized the lethal, hemorrhagic, myotoxic, and phospholipase A_2_ activities induced by *B. brazili* venom. The reactivity of *Bothrops* antivenom with *B. brazili* and *B. b. smaragdinus* was also confirmed via antivenomics [23,24]. The commercial antivenom showed paraspecific immunoreactivity against all the toxin classes of *B. b. smaragdinus* and *B. brazili* venoms, with maximal binding to PI- and PIII-class SVMPs but diminished reactivity against *B. brazili* PLA_2_s, which comprise the major toxin class of the *B. brazili* [23]. In addition, Muniz et al. [68] reported that the Brazilian *Bothrops* antivenom neutralized the lethal activity of venoms from *B. brazili*, *B. taeniatus*, *B. atrox*, and *B. b. smaragdinus*, also supporting our data. Taken together, these data suggest that Brazilian *Bothrops* antivenom can be used against the species collected in the Amazon, supporting its efficacy in the treatment of human accidents involving those rare species of bothropoids.

## 3. Conclusions

Venom composition can be considered a trophic adaptation in some groups of snakes and has become an important model system for understanding ecological and evolutionary processes that underlie adaptive traits. The toxic arsenal comprised in snake venoms is mostly composed of proteins that belong to a few protein families [69], which are represented by several different isoforms with variable proportions [31]. The differences in isoform presence and proportion modulate the venom function [70]. Therefore, it is tempting to assume that the complexity of snake venoms is ecologically shaped, and it is crucial for the foraging success. Moreover, snake venom complexity does not appear to be phylogenetically constrained [71], but evolves in association with the diversity of diet items [72]. Also, ecological pressures have been associated with factors driving optimal solutions that resulted in the combination of a few major toxin families [73]. The evolution of this optimal set of toxins resulted in the snake venom variability found at different levels, like populational, geographic, and ontogenetic levels. In this study, we characterized the venom composition of five bothropoids. Our results highlighted substantial differences among venom composition; however, they did not offer any evidence that venom composition can be associated with different habitats (arboreal vs. terrestrial), or abundance (abundant vs. rare). Comparing species with the same habitat, the venom of *B. taeniatus* appeared to be more complex than the venom of *B. b. smaragdinus*, but the latter presented SVMP and PLA_2_ isoforms related to the most striking activities of the venoms of *Bothrops* snakes: the myotoxic K49-PLA_2_ [49] and the jararhagin-like SVMP [48]. On the other hand, *B. b. smaragdinus* shared a similar composition with the mainly terrestrial *B. atrox*, including the enzymatic activities and the presence of relevant isoforms. Another point to be considered is the role of venom toxicity in shaping the success of a species occupying different ecological niches. *Bothrops atrox* is the most abundant venomous snake in the Amazonian territory; however, it apparently presents one of the simplest and least lethal venoms. Therefore, our results tend to indicate that venom composition might not shape the species’ ecological constraints. Consequently, other biotic (e.g., competition and prey availability) or abiotic factors (e.g., temperature and humidity) may have a greater influence on snake foraging habits and dispersal capacities than the composition or toxicity of their venoms.

## 4. Materials and Methods

### 4.1. Snakes and Venoms

Two specimens of *B. hyoprora*, three specimens of *B. b. smaragdinus*, three specimens of *B. brazili*, three specimens of *B. atrox*, and one specimen of *B. taeniatus* were collected in the Alto Juruá region, in the municipalities of Mâncio Lima and Sena Madureira, Acre State of Brazil, and Guajará, Amazonas State of Brazil, under ICMBio/SISBIO permits 12178, 66367, and 65653. Sex and snout-vent length (SVL) were annotated for each specimen and are depicted in Table 2. Venom samples were collected using manual extraction techniques soon after snake capture, without any previous feeding at the lab. Venom samples were individually freeze-dried and kept at −80 °C until used. For proteomics and functional tests, venoms were resuspended in PBS, protein quantities estimated with OD 280 nm, and equal amounts of venom proteins mixed to perform the pool of venom representing each species. Four days after venom extraction, venom glands were dissected and immediately transferred to RNALater (Invitrogen, Life Technologies Corp.) and stored at −80 °C until RNA extraction and mRNA isolation.

### 4.2. Venom Fractionation by Reverse Phase Chromatography

Venom samples (2 mg) dissolved in 500 mL 0.1% trifluoroacetic acid (TFA) were fractionated on a reverse-phase column (Jupiter Phenomenex, 10 µm C18 300 Å, 250 × 10 mm) via HPLC (Shimadzu, Japan), as previously described [9]. The parameters for elution were 2 mL/min flow, with a gradient of 0.1% TFA in water (solution A) or in acetonitrile (solution B) as follows: 5% B for 5 min, followed by 5 to 15% B over 10 min, 15 to 45% B over 60 min, 45 to 70% B over 10 min, 70 to 100% B over 5 min, and 100% B over 10 min. Detection was performed with UV at 214 nm.

### 4.3. Transcriptome

#### 4.3.1. RNA Extraction and Analysis; cDNA Library Construction and Sequencing

The RNA was extracted as previously described [74]. RNA extraction, quantification, quality control, and library preparation were carried out as previously described [75]. Briefly, libraries were prepared with 1 µg of each sample with an Illumina TruSeq Stranded RNA HT kit consisting of TruSeq Stranded RNA HT/cDNA Synthesis PCR, TruSeq Stranded RNA HT/Adapter Plate Box, and TruSeq Stranded HT mRNA. Fragment size distributions were evaluated via microfluidic gel electrophoresis using an Agilent DNA 1000 Kit. Quantification of each library was then performed with real-time PCR using a KAPA SYBR FAST Universal qPCR Kit, using the StepOnePlus Real-Time PCR System. Aliquots of each cDNA library were diluted to a concentration of 2 nM. The cDNA libraries were sequenced on an Illumina HiSeq 1500 System in Rapid Run mode using a paired-end flow cell for 300 cycles of 2 × 150 bp.

#### 4.3.2. Transcriptome Assembly and Annotation

To assemble the venom transcriptomes of the samples, we used an in-house script [35], trimmed the sequencing adaptors using TrimGalore [76], and merged the paired reads using PEAR software v0.9.8 [77] by the common overlap on the 3′ ends [34]. We used five different assemblers with different k-mer values and assembly parameters (Trinity: k-mer 31; rnaSPADES: k-mer 31, 75, and 127; Extender: default, overlap 150, and seed size 2000; SeqMan Ngen: k-mer 21; and Bridger: k-mer 30) [34,35,78,79,80]. Coverage analysis and annotation were performed as described in [75]. The coding sequences annotated from all individuals of the same species were grouped into a single file at 98% identity using cd-hit-est [35,81] and the expression of each transcript was estimated in transcripts per million (TPM) using the RSEM software v1.3.3 [81] after mapping the merged reads from each sample using Bowtie2. Raw transcriptomic data are available at NCBI’s GenBank under Bioproject accession number PRJNA 1040254 and Biosamples accession number SAMN38250442 to 38250451.

### 4.4. Proteomic Analysis of Venoms

#### 4.4.1. Analysis via In-Solution Trypsin Digestion and LC−MS/MS

Proteomic analysis was carried out with 30 μg of pooled venom samples from each species according to the methods currently used in our laboratory [82] with small modifications. Proteins were digested with trypsin using the Filter Aided Sample Preparation (FASP) [83], and the peptide mixtures were desalted using the stage tip approach [84] with C18 SDB-XC membranes (Empore^TM^) (Thermo Scientific, Waltham, MA, USA). LC−MS/MS analyses were performed in a Vanquish Neo (Thermo Scientific, Waltham, MA, USA) UPLC system coupled to an Orbitrap Exploris 480™ mass spectrometer (Thermo Fisher Scientific, Waltham, MA, USA). Samples were injected in a PepMap™ Neo (Thermo Scientific) precolumn containing 5 mm of 5 µm C18 beads, and the chromatographic separation of the peptides was performed in a PepMap™ Neo analytical column (Thermo Scientific) containing 150 mm of 2 µm C18 beads using a gradient of acetonitrile/0.1% formic acid with steps of 2–45% in 55 min, 45–99% in 0.1 min, and 99% for 5 min, under a flow of 300 nL/min. The mass spectrometer was adjusted to operate at 2 kV in positive mode and the transfer tube temperature was set to 270 °C, operated in Data Dependent Acquisition, using high-energy collision-induced dissociation, with a resolution of 120,000 for MS1 fragments, with a normalized AGC target of 300%, maximum inject time was set to auto, and 15,000 for MS2 fragments. The scanning range was established for the window of 200–2000 *m*/*z* and positive charge states of 2–5. Mass spectrometry data were deposited via the PRIDE [85] repository. Data are available via ProteomeXchange with identifier PXD046872.

#### 4.4.2. Identification and Relative Quantification

Raw data were processed and searched using the search tool Peaks (Version 10.5) against an in-house database consisting of the protein sequences predicted from a nonredundant transcriptome master set of the same specimens used for venom extraction, using the same parameters as previously described [75]. Scaffold (version Scaffold_5.2.1) was used to validate MS/MS-based peptide and protein identifications. Peptide identifications were accepted if they could be established at greater than 95.0% probability via the Peptide Prophet algorithm [86] with Scaffold delta-mass correction. Protein probabilities were assigned with the Protein Prophet algorithm [87]. The Normalized Spectral Abundance Factor (NSAF) quantitative method was used to estimate the general abundance of independent isoforms and toxin groups.

### 4.5. Sequence Alignments and Gene Tree Analyses

Complete cDNA sequences were translated into their predicted proteins (expasy.ch, translate tools) and subjected to phylogenetic constructions partitioned into four groups according to their toxin groups: 1—SVMPs; 2—SVSPs; 3—PLA_2_; and 4—CTLs. In each group, we included protein sequences to root the trees: for SVMPs, ADAM 28 from Mus musculus (NCBI_NP_899222.1); for SVSPs, the thrombin-like toxin from Ophiophagus Hannah (GenBank: ABN72544.1); for PLA_2_, a PLA sequence from Ophiophagus Hanna (GenBank: AAG17443.1); and for CTL, a C-Type lectin from Pseudonaja Textilis (GenBank: ABP94087.1). Trees were assembled using the NGPhylogeny.fr interface [88,89]. Protein sequences were aligned with the iterative refinement method E-INS-i using a BLOSUM62 scoring matrix as implemented in MAFFT 7.407_1 [90], and alignment curation was performed via BMGE 1.12_1 [91]. Maximum likelihood (ML) phylogenetic trees were estimated using PhyML 3.3_1 [92,93] and rendered with Newick Display 1.6 [94] and iTOL 6.8_1 [95].

### 4.6. Enzymatic Assays

SVMP, SVSP, and PLA2 enzymatic activities of pooled venoms from each species were assayed using synthetic substrates as previously described [9]. The results represent the mean ± SD of three independent experiments, undertaken in triplicate.

### 4.7. Reactivity with Antivenoms

The reactivity of the Bothrops antivenom with the venoms from the different species was evaluated with ELISA and Western blotting as previously described [71].

### 4.8. Lethality Test

The lethality induced by the venoms was tested in Swiss mice with 18–20 g body weight with protocols approved by the Animal Ethical Use Committees of the Instituto Butantan (CEUA nº 4222230223—ID 003103). Groups of 5 mice were injected intraperitoneally with a single dose (300 μg) of venom samples, diluted in 500 μL of PBS. Following injection, the times of death for each group were recorded at 30, 60, 90, 150, 180, 240, and 360 min, and then at 12, 24, and 48 h after inoculation of the samples. After 48 h, the surviving animals were euthanized, using a CO_2_ chamber. For neutralization tests, venoms (300 μg) were preincubated with 240 μL *Bothrops* antivenom at 37 °C for 30 min, centrifuged at 12,000 rpm, and the supernatant was used in the tests as described above. The data obtained were graphed on a survival curve plot in a linear scale up to 6 h, as no death was recorded after this time.

## Figures and Tables

**Table 1:** Number of toxin transcripts recovered from venom gland transcriptome of each species transcriptome grouped according to the toxin classes.

Toxin Class *	*Bothrocophias hioprora*	*Bothrops atrox*	*Bothrops bilineatus smaragdinus*	*Bothrops brazili*	*Bothrops taeniatus*
SVMP	14	6	17	14	12
CTL	4	16	22	9	15
SVSP	12	6	12	13	7
PLA_2_	2	2	5	3	4
VEGF	3	2	3	3	2
LAO	0	2	1	1	0
HYAL	1	1	1	1	0
CRISP	1	1	1	1	1
NGF	1	1	1	1	0
NUC	1	0	1	1	2
PDE	2	1	2	2	1
PLB	1	1	1	1	1
BPP	1	0	1	1	0
DIPEP	1	0	1	1	0
CYS	1	0	1	2	0
iPLA	0	0	1	1	0
LIPA	1	0	1	1	1
WAP	1	0	0	2	1
KUN	1	0	0	0	0
Total	48	39	72	58	47

* SVMP—Snake Venom Metalloproteinases; CTL—C-Type Lectin-Like Toxins; SVSP—Snake Venom Serine Proteinases; PLA_2_—Phospholipases A2; VEGF—Vascular Endothelial Growth Factors; LAO—L-Aminoacid Oxidases; HYAL—Hialuronidases; CRISP—Cysteine Rich Secretory Proteins; NGF—Nerve Growth Factors; NUC—5′Nucleotodases; PDE—Phosphodiesterases; PLB—Phospholipases B; BPP—Bradikinin Potenciating Peptides; DIPEP—Dipeptidyl Peptidases; CYS—Cystatin; iPLAs—Phospholipase inhibitors; LIPA—Lipases; WAP—Warprins; and KUN—Kunitz Trypsin Inhibitor.

**Table 2:** Description of the snake specimens used in this study.

Code	Genus	Sex	Age	SVL (mm)	City	Locality
SB0779	*Bothrocophias hyoprora*	F	adult	370	Mâncio Lima—Acre	PARNA Serra do Divisor, Formosa trail
SB0780	*Bothrocophias hyoprpra*	M	juvenile	252	Mâncio Lima—Acre	PARNA Serra do Divisor, Formosa trail
SB0597	*Bothrops atrox*	M	adult	1020	Sena Madureira—Acre	RESEX Cazumbá-Iracema
SB0600	*Bothrops atrox*	F	adult	1090	Sena Madureira—Acre	RESEX Cazumbá-Iracema
SB0796	*Bothrops atrox*	M	adult	1035	Mâncio Lima—Acre	PARNA Serra do Divisor, Formosa trail
SB0582	*Bothrops b. smaragdinus*	M	adult	532	Sena Madureira—Acre	RESEX Cazumbá-Iracema
SB0782	*Bothrops b. smaragdinus*	F	adult	675	Mâncio Lima—Acre	PARNA Serra do Divisor, Formosa trail
SB1942	*Bothrops b. smaragdinus*	M	adult	534	Mâncio Lima—Acre	PARNA Serra do Divisor, Formosa trail
SB1455	*Bothrops brazili*	M	adult	795	Mâncio Lima—Acre	Serra do Divisor
SB1948	*Bothrops brazili*	F	adult	685	Mâncio Lima—Acre	PARNA Serra do Divisor, Formosa trail
SB1953	*Bothrops brazili*	F	juvenile	341	Guajará—Amazonas	ND
SB1580	*Bothrops taeniatus*	F	adult	517	Mâncio Lima—Acre	PARNA Serra do Divisor

## Data Availability

Proteomics raw data are available via ProteomeXchange with identifier PXD046872. Raw transcriptomic data are available at NCBI’s GenBank under Bioproject accession number PRJNA1040254 and Biosamples accession number at SAMN38250442 to 38250451.

## Supplementary Materials

The following supporting information can be downloaded at: https://www.mdpi.com/article/10.3390/toxins16020083/s1, Supplementary Table S1. Transcriptomic data of the specimens used in this study; Supplementary Table S2. Tryptic peptides of *B. taeniatus*, *B. bilineatus smaragdinus*, *B. atrox*, *B. brazili*, and *Bothrocophyas hyoprora* venoms identified with LC-MS/MS.
